# Near vision in patients with DME and RVO treated with aflibercept and correlation with NEI VFQ-25 questionnaire

**DOI:** 10.1186/s40942-024-00558-0

**Published:** 2024-05-23

**Authors:** Zuzana Anwarzai Sulavikova, Zuzana Sustykevicova, Marek Kacerik, Vladimir Krasnik

**Affiliations:** 1 Department of Ophthalmology, Faculty hospital in Trencin, Trencin, Legionarska 28, 91101 Slovakia; 2 Department of Ophthalmology, Comenius University and University hospital in Bratislava, Bratislava, Slovakia

**Keywords:** Near vision, NEI VFQ-25, Aflibercept, DME, RVO

## Abstract

**Background:**

The aim of this study is to evaluate near and distance visual acuity (VA) and their correlation with the National Eye Institute Visual Function Questionnaire (NEI VFQ-25) outcomes in patients with diabetic macular edema (DME) and macular edema due to retinal vein occlusion (RVO) treated with aflibercept.

**Methods:**

In this prospective study, we included 87 eyes of patients diagnosed with DME (*n* = 61) and RVO (*n* = 26), who received aflibercept treatment and were followed until the 8th injection. Near VA was examined on the 1st, 2nd, 3rd, 4th, 6th, and 8th injection, and patients completed the NEI VFQ-25 on the 1st, 4th, and 8th aflibercept injection.

**Results:**

The mean near VA at baseline in all eyes was 0.89 ± 0.12 logMAR. With every administration, there was a statistically significant improvement; on the 4th (0.70 ± 0.19; *p* = 0.000) and the 8th application (0.60 ± 0.19; *p* = 0.000). At baseline, the mean NEI VFQ-25 total score was 71 ± 14%, and improved to 81 ± 13% (*p* = 0.000) on the 8th injection. The most significant score gain was recorded in the near VA subscale (+ 20 ± 14%, *p* = 0.000). There was no statistically significant difference between DME and RVO group in the questionnaire or near VA outcomes.

**Conclusion:**

Aflibercept treatment resulted in a remarkable improvement of near vision by 4 lines of logMAR optotype after the 8th application. The near vision questionnaire subscale, initially scoring the lowest, exhibited the greatest gain during the treatment period. This underscores the importance of near vision and reading ability for patients with DME and RVO.

## Background

Diabetic macular edema (DME) and retinal vein occlusion (RVO) stand out as prevalent vascular retinal conditions. The impact of aflibercept on distance best-corrected visual acuity (BCVA) has been substantiated by numerous clinical trials [1–4]. BCVA is routinely monitored during every patient visits and serves as a key criteria for treatment evaluation in both clinical trials and also in practice [1–4].

When assessing near visual acuity (VA), a larger anatomical and functional area of the macula is involved, testing approximately a 20° field of view. In BCVA examinations, the functionality of the 1° central retina is tested, requiring a smaller area for reading a single character on an optotype. Rayner reported that when testing near vision, a visual field extending from the currently fixated word to 15 characters to the right of fixation is necessary. Observing near VA can provide more comprehensive insight into the anatomical and functional state of the macula [5, 6].

Reading difficulties often manifest as an initial symptom of macular disorders. However, near VA is not routinely examined in patients with DME and RVO. To our knowledge and based on our research, no studies have investigated the benefits of near vision in DME and RVO eyes treated with aflibercept. Limited data available regarding near vision in eyes with age-related macular degeneration (AMD) following anti-vascular endothelial growth factor (anti-VEGF) treatment [6–8].

Most clinical trials for assessing near vision use the National Eye Institute Visual Function Questionnaire (NEI VFQ-25), which comprises 25 questions for the subjective assessment of various visual functions and daily vision-dependent activities, including near vision [9, 10]. This questionnaire is not commonly used in routine practice due to its time-consuming and comprehensive nature. Notably, none of the clinical trials for DME and RVO have correlated near and distance VA after intravitreal injection with NEI VFQ-25 outcomes.

The primary aim of this study is to evaluate real-world outcomes of near VA, BCVA, optical coherence tomography (OCT), and their correlation with patients’ subjective NEI VFQ-25 scores in individuals with DME and RVO treated with 8 injections of 2.0 mg/0.05 ml aflibercept.

## Methods

Between January 2021 and January 2024, a total of 87 eyes from 68 patients diagnosed with DME and RVO were enrolled in this prospective study. The study was conducted at the Department of Ophthalmology, Faculty Hospital Trencin in Slovakia. The follow-up period extended until the 8th aflibercept application. All enrolled eyes were newly diagnosed with previously untreated DME or RVO. Exclusion criteria included ocular surgery or laser photocoagulation during the follow-up period or within six months prior to enrolment, as well as other severe eye disorders significantly limiting visual acuity.

Patients with DME received five initial injections at four-week intervals, followed by intravitreal aflibercept 2 mg every eight weeks in the first year. Patients with RVO received the first three injections at four-week intervals, and then the treat-and-extend regimen was applied. Due to the varying treatment regimens, we present the cohort’s timeline in the number of injections rather than weeks of treatment.

Distance BCVA was measured using the Early Treatment of Diabetic Retinopathy Study (ETDRS) optotype from a distance of 4 m at every visit. Central retinal thickness (CRT) in µm was obtained on every visit using Heidelberg Spectralis OCT (Heidelberg Engineering, Heidelberg, Germany).

Near VA was examined on the 1st, 2nd, 3rd, 4th, 6th, and 8th injection days. A printed optotype with a logarithmic progression was used from a distance of 40 cm in logMAR. The reading distance was measured using a red string that is part of the optotype. During the examination, + 3 diopters were added to the correction obtained from BCVA and corrected individually. Monocular examination was performed, with the fellow eye occluded.

Patients completed the NEI VFQ-25 questionnaire version 2000 on the 1st, 4th, and 8th applications before mydriatics administration. The questionnaire, in the Slovak language, was filled out in the presence of a doctor or nurse. Patients were given privacy and adequate time to understand the questionnaire, and any additional questions were answered. Scoring of the questionnaire was done manually following the NEI VFQ-25 scoring algorithm (version August 2000). The general health subscale was not included in the total score. Initially, we evaluated the results of all eyes together and then divided the group according to diagnosis into DME and RVO. All the mentioned procedures were carried out in the following order: BCVA and near vision assessment, NEI VFQ-25 questionnaire administration, OCT imaging, and finally, intravitreal injection was performed.

Statistical analyses included independent t-tests, chi-squared tests, and paired t-tests to assess differences between groups. A *p*-value less than 0.05 was considered statistically significant. Pearson’s test was utilized to examine correlations between questionnaire results (total score or subscale score) and near VA, BCVA, or OCT measurements. Quantitative variables (e.g., age, NV) were analysed using descriptive statistics, including the count of measurements, mean, and standard deviation. Qualitative variables were analysed using absolute and relative frequencies. Differences between groups in qualitative variables were evaluated using the independent samples t-test and the Chi-square test of independence. Changes in qualitative variable values between two time points were assessed using paired t-tests. The study adhered to the principles outlined in the Declaration of Helsinki.

## Results

A total of 87 treatment-naïve eyes from 68 Caucasian patients were enrolled. Nineteen patients had follow-ups with both eyes. 61 eyes (70%) were diagnosed with DME, and 26 eyes (30%) were diagnosed with RVO, including 15 (17%) with branch RVO and 11 (13%) with central RVO. The mean age of patients at baseline was 66.8 ± 9.3 (33–87) years, with 52 (60%) being men. Among the total 87 eyes, 51 (59%) were phakic, and 36 (41%) were pseudophakic eyes with a monofocal implant (Table 1). The average glycated haemoglobin value in patients with diabetes mellitus was 7.6 ± 0.7% (5.9–9.9) DCCT, with 54 eyes (89%) treated for diabetes type 2 and 7 eyes (11%) treated for type 1. Patients with RVO (69.6 ± 9.9 years) were older than patients with DME (65.6 ± 8.9 years), but the age difference did not reach statistical significance (*p* = 0.067). Complete follow-up until the 8th application of aflibercept was achieved by 59 (68%) of the original 87 eyes, including 19 (73%) with RVO and 40 (66%) with DME eyes. The early termination of follow-up before the 8th injection occurred in 28 (32%) eyes for various reasons, such as improvement (3%), death (1%), elevated glycated haemoglobin (11%), non-responder requiring a switch (10%), pars plana vitrectomy for epiretinal membrane (2%), and unknown reasons (4%).

**Table 1:** Basic demographic characteristics of study group

Study group	*n* = 87
DME eyes	*n* = 61 (70%)
RVO eyes	*n* = 26 (30%)
Mean age (years)	66.8 ± 9.3 (33–87)
Male: female	52(60%) : 35(40%)
Fakic: pseudofakic eye	51(59%) : 36(41%)
Right : left eye	40(46%) : 47(54%)

### BCVA outcome

**Fig. 1:**
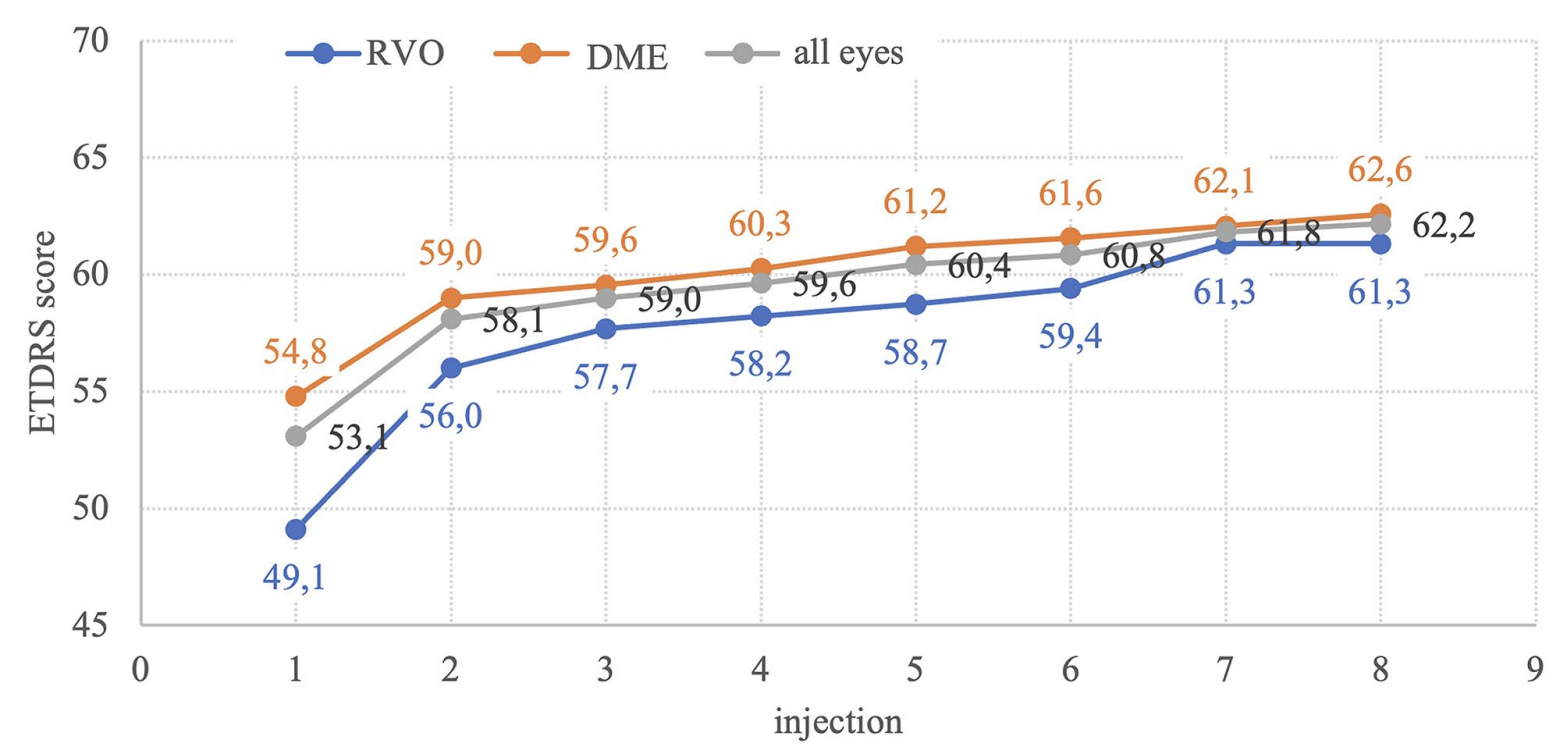
Distance best corrected visual acuity outcomes (ETDRS) are presented for the entire eyes cohort in grey, retinal vein occlusion (RVO) eyes in blue, and eyes with diabetic macular edema (DME) in orange

The mean BCVA of all patients on the 1st injection was 53.1 ± 12 ETDRS letters. With each subsequent injection, there was a statistically significant improvement in ETDRS score (Fig. 1). On the 4th application, there was an improvement to 59.6 ± 12, and on the 8th, it increased further to 62.2 ± 12 ETDRS letters. Statistically significant improvements were also found when dividing eyes into the DME group (54.8 ± 11; 60.3 ± 11; 62.6 ± 13) and the RVO group (49.1 ± 13; 58.2 ± 13; 61.3 ± 12). When comparing the BCVA results between the RVO and DME groups, a statistically significant difference (*p* = 0.042) was confirmed between the average ETDRS value on the 1st application for eyes with DME (55 ± 11) and those with RVO (49 ± 13). When comparing the letter gain on the 2nd, 3rd, and 4th application between subgroups RVO (+ 6.9 ± 6.1; +8.6 ± 8.4; +9.1 ± 7.4) and DME (+ 4.2 ± 4.5; +4.8 ± 6.0; +5.8 ± 5.6), a significant difference was found (*p* = 0.052; *p* = 0.019; *p* = 0.045).

### OCT outcome

**Fig. 2:**
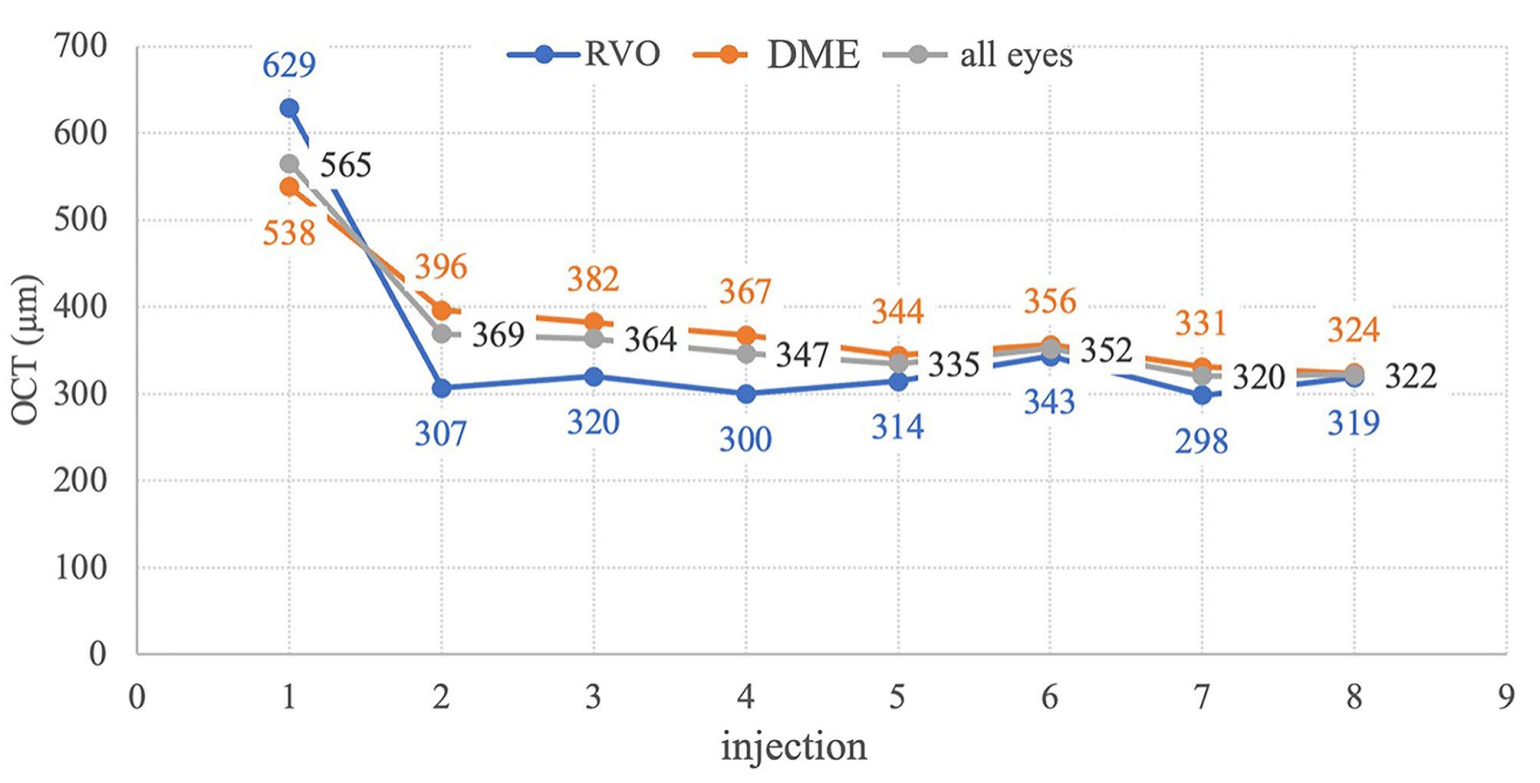
Central retinal thickness is presented for the entire eyes cohort in grey, retinal vein occlusion (RVO) eyes in blue, and eyes with diabetic macular edema (DME) in orange

The mean CRT in all eyes at the 1st aflibercept injection was 565 ± 151 μm (Fig. 2). With each subsequent application (up to the 8th application), there was a statistically significant decrease. Specifically, on the 4th application, there was a significant decrease to 347 ± 92 μm, and on the 8th to 322 ± 95 μm in the entire cohort (*p* < 0.001 for comparisons with the 1st injection). Statistically significant improvements were also found when dividing the eyes into DME (538 ± 127; 367 ± 83; 324 ± 90) and RVO (629 ± 185; 300 ± 95; 319 ± 107) µm. When comparing the CRT results between the RVO and DME groups, a statistically significant difference (*p* = 0.029; *p* = 0.000; *p* = 0.009; *p* = 0.001) was confirmed between the average CRT on the 1st, 2nd, 3rd, and 4th application in eyes with DME (538 ± 127; 399 ± 94; 382 ± 91; 367 ± 83) and in RVO eyes (629 ± 185; 307 ± 74; 320 ± 117; 300 ± 95).

### Near vision outcome

**Fig. 3:**
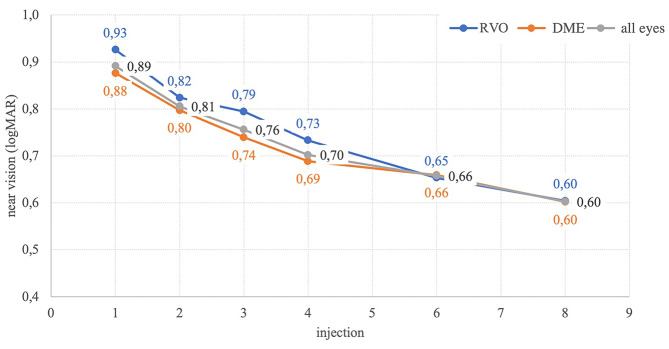
Near visual acuity outcomes (logMAR) are presented for the entire eyes cohort in grey, retinal vein occlusion (RVO) eyes in blue, and eyes with diabetic macular edema (DME) in orange

**Fig. 4:**
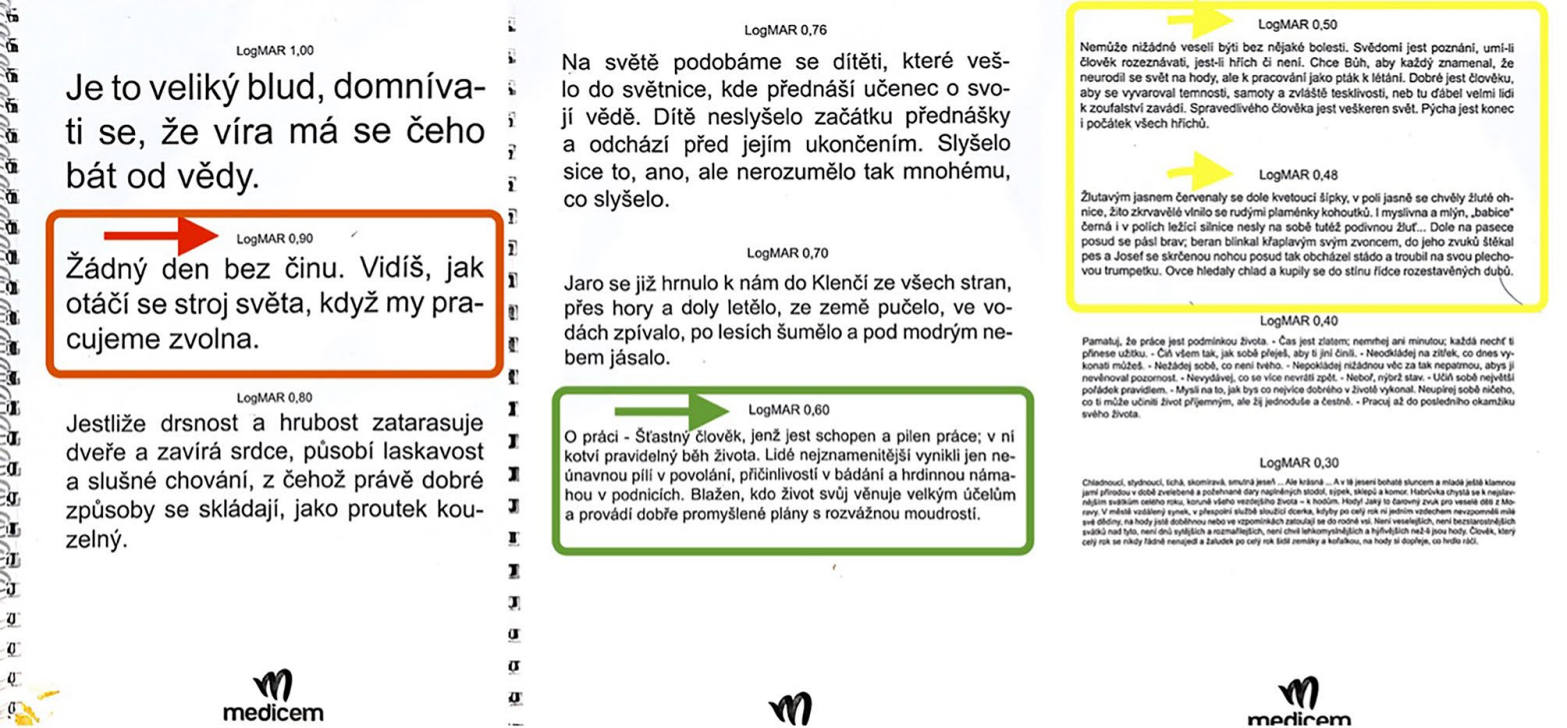
The scanned near vision chart used in the study. Baseline near visual acuity averaged 0.89 ± 0.12 (red), demonstrating improvement to 0.60 ± 0.19 logMAR by the 8th injection (green). The logMAR range of 0.48–0.50 (yellow) corresponds to the text size typically found in books

Fig. 3; Table 2 displays the mean near VA and the change compared to the 1st injection with its statistical significance. The average near VA on the 1st application in all eyes was 0.89 ± 0.12 logMAR. With each subsequent application (up to the 8th injection), there was a statistically significant decrease. Specifically, on the 4th application, there was a significant decrease to 0.70 ± 0.19 logMAR (*p* = 0.000), and on the 8th, it decreased further to 0.60 ± 0.19 logMAR (*p* = 0.000). Significant improvements were found in the distribution of eyes according to diagnosis on the 1st, 4th, and 8th applications in the DME group (0.88 ± 0.12; 0.69 ± 0.18; 0.60 ± 0.17) and RVO group (0.93 ± 0.11; 0.73 ± 0.21; 0.60 ± 0.22) logMAR. In contrast to the OCT and BCVA values, no statistically significant difference in near vision values was confirmed between DME and RVO eyes during follow-up. Thus, changes in near VA during treatment were similar between RVO and DME. In simple terms, we could say that after the 8th aflibercept injection, near vision improved by approximately 4 lines of our optotype (Fig. 4). This improvement applies to the entire cohort and also when divided by diagnosis into DME and RVO. Font sizes logMAR 0.48 and 0.50 correspond to the font in books and magazines. This and a better (lower) value were achieved in 33 eyes during the follow-up.

**Table 2:** Complete near vision outcomes with statistical significance

DME + RVO						
	1st	2nd	3rd	4th	6th	8th
Measurements	*n* = 87	*n* = 87	*n* = 87	*n* = 85	*n* = 65	*n* = 59
Mean near VA, logMAR ± SD	0.89 ± 0.12	0.81 ± 0.16	0.76 ± 0.17	0.70 ± 0.19	0.66 ± 0.19	0.60 ± 0.19
Baseline improvement		-0.09 ± 0.09	-0.14 ± 0.10	-0.19 ± 0.13	-0.24 ± 0.14	-0.29 ± 0.14
*P*-value		0.000	0.000	0.000	0.000	0.000
**DME**						
Measurements	*n* = 61	*n* = 61	*n* = 61	*n* = 59	*n* = 44	*n* = 40
Mean near VA, logMAR ± SD	0.88 ± 0.12	0.80 ± 0.16	0.74 ± 0.17	0.69 ± 0.18	0.66 ± 0.18	0.60 ± 0.17
Baseline improvement		-0.08 ± 0.08	-0.14 ± 0.09	-0.19 ± 0.12	-0.23 ± 0.12	-0.28 ± 0.11
*P*-value		0.000	0.000	0.000	0.000	0.000
**RVO**						
Measurements	*n* = 26	*n* = 26	*n* = 26	*n* = 26	*n* = 21	*n* = 19
Mean near VA, log MAR ± SD	0.93 ± 0.11	0.82 ± 0.17	0.79 ± 0.17	0.73 ± 0.21	0.65 ± 0.20	0.60 ± 0.22
Baseline improvement		-0.10 ± 0.11	-0.13 ± 0.12	-0.19 ± 0.16	-0.26 ± 0.17	-0.30 ± 0.20
*P*-value		0.000	0.000	0.000	0.000	0.000

### NEI VFQ-25 outcomes

Table 3 shows mean score outcomes (total score + 12 subscale scores) of the NEI VFQ-25 questionnaire on the 1st, 4th, and 8th aflibercept injections in the entire cohort. On the 1st injection, the mean total questionnaire score was 71 ± 14%. On the 4th application, the mean total questionnaire score increased to 78 ± 14% (*p* = 0.000), and on the 8th, it improved to 81 ± 13% (*p* = 0.000). On the 8th injection, there was a statistically significant improvement in the total score and also in all vision-related subscale scores. When analysing subscale scores, the most significant gain occurred in the near vision subscale (gain + 20 ± 14%, *p* = 0.000) and distance vision subscale (+ 15 ± 13%, *p* = 0.000). Also, the near vision subscale in the NEI VFQ-25 questionnaire had the lowest score at baseline and reached the greatest score gain during treatment. This means that at baseline, patients subjectively rated the decrease in near vision the worst, and at the same time, during treatment, this aspect improved subjectively the most. No significant differences were found between DME and RVO eyes.

**Table 3:** Mean score outcomes of NEI VFQ-25 questionnaire on the day of 1st, 4th and 8th aflibercept injection in all eyes

	1st	4th	8th
Measurements	*n* = 86	*n* = 84	*n* = 59
Total score	71 ± 14	78 ± 14 *p* = 0.000	81 ± 13 *p* = 0.000
General health	43 ± 15	40 ± 15 *p* = 0.032	46 ± 15 *p* = 0.057
General vision	58 ± 18	65 ± 16 *p* = 0.000	68 ± 16 *p* = 0.000
Ocular pain	71 ± 16	81 ± 19 *p* = 0.000	85 ± 17 *p* = 0.000
Near vision	**53 ± 18**	**66 ± 20 ** ***p*** ** = 0.000**	**72 ± 21 ** ***p*** ** = 0.000**
Distance vision	**66 ± 19**	**77 ± 17 ** ***p*** ** = 0.000**	**78 ± 17 ** ***p*** ** = 0.000**
Social functioning	77 ± 18	81 ± 16 *p* = 0.000	84 ± 14 *p* = 0.000
Mental health	69 ± 20	73 ± 19 *p* = 0.001	75 ± 17 *p* = 0.000
Role difficulties	64 ± 21	73 ± 19 *p* = 0.000	74 ± 20 *p* = 0.000
Dependency	84 ± 22	87 ± 21 *p* = 0.122	88 ± 21 *p* = 0.000
Driving	83 ± 14	88 ± 12 *p* = 0.044	93 ± 12 *p* = 0.000
Color vision	88 ± 16	94 ± 12 *p* = 0.000	95 ± 10 *p* = 0.000
Peripheral vision	74 ± 18	80 ± 19 *p* = 0.000	83 ± 17 *p* = 0.000

**Fig. 5:**
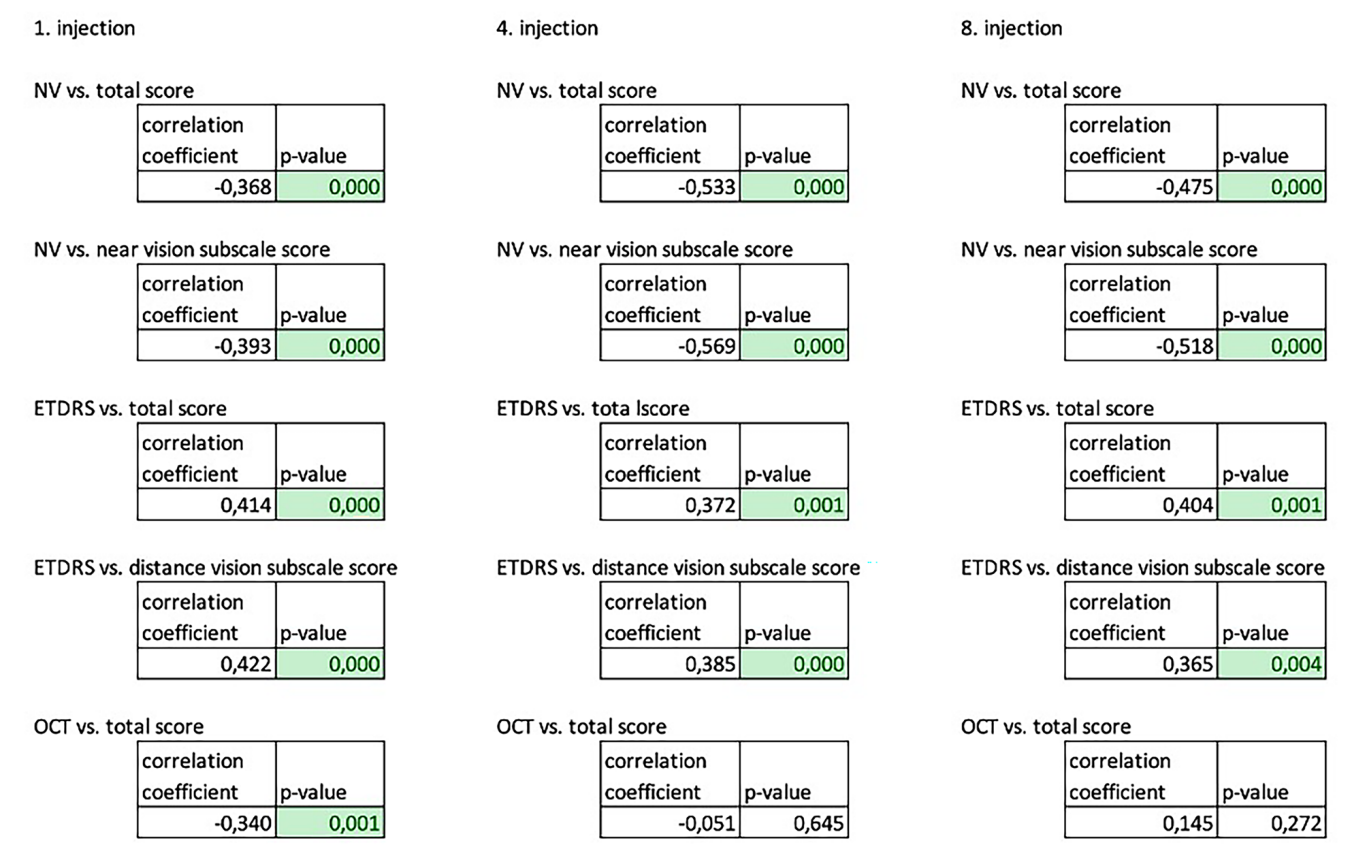
Correlations between near vision, OCT, BCVA, and questionnaire score outcomes are illustrated *OCT-optical coherence tomography, ETDRS-Early Treatment of Diabetic Retinopathy Study, NV-near vision*

We verified the correlations with the Pearson test based on a positive or negative correlation coefficient and statistical significance. Fig. 5 displays all possible correlations with their correlation coefficient and statistical significance. We confirmed a statistically significant correlation between the values of near VA with the total score and with the near VA subscale score. Correlation was also confirmed between BCVA and the total score, as well as the distance vision subscale score. On the contrary, we did not confirm the correlation between CRT changes on OCT and the total score of the questionnaire on the 1st, 4th, and 8th injections in the whole cohort. It means that the values of BCVA and near VA are directly correlated with the questionnaire score.

## Discussion

Our study, breaking new ground by assessing both near and distance visual acuity (VA) and their correlation with NEI VFQ-25 scores in patients with DME and RVO. We found near VA examinations to be practical, quick, and reproducible, reinforcing their importance based on patient feedback. Notably, after 8 aflibercept applications, near VA improved by approximately 4 lines on the logMAR optotype.

Literature on anti-VEGF effects on near vision in DME and RVO is sparse. Limited data from AMD studies showed varying near VA improvements. Frennesson followed near VA in 30 patients after 3 ranibizumab injections with Tomteboda optotype (Tomteboda Resource Center for Visually Handicapped Children, Stockholm, Sweden) graded with points, where the smallest text had 4 and the largest 24 points. Frennesson reported an improvement from 9 ± 5 points to 6 ± 3 points after 3 ranibizumab injections [7]. Epstein followed 85 patients with AMD treated with aflibercept for 18 months. In these patients, there was a more significant improvement in near VA compared to BCVA. They used a similar optotype with the same scoring point system and the average near VA improved from 12 to 5 points in the first year, maintaining this value until the 18th month [6]. Our previous study with logMAR optotype on 30 AMD patients treated with aflibercept at the Faculty Hospital in Trencin showed a gradual improvement in near VA from 0.63 to 0.44 logMAR [8].

In our search, no publications were found for DME/RVO eyes for direct comparison of near VA results. Rajasekar monitored near VA in 148 DME eyes. In phakic group (*n* = 72), there were 65 eyes (90.3%) with stable/improved near vision and 59 eyes (81.9%) with stable/improved distance vision, whereas in the pseudophakic group (*n* = 76), 63 eyes (82.9%) and 60 eyes (78.9%), respectively. Only near vision improvement in both phakic and pseudophakic eyes showed 7.7–13% of the cohort. This results were not statistically significant considering that most patients received only 1 injection, just 14% of eyes received more than 3 injections and also around 87% of patients were treated with bevacizumab and rest of eyes with ranibizumab [11].

The NEI VFQ-25, a validated instrument for assessing vision-related quality of life, has been employed in various clinical studies [12]. Our study, analysing questionnaire results statistically and comparing them with other studies, demonstrated an improvement of + 11 ± 8% in vision-related quality of life after the 8th aflibercept. The near vision subscale score showed the most significant gain at + 20 ± 14%, emphasizing the subjective importance of near vision. No significant difference was found between DME and RVO scores. We compared our results of DME eyes (*n* = 61) from the 8th application with the results in AQUA study from the week 52 (8.8 aflibercept injection). At baseline, the average total score was 70.12% and the mean BCVA was 61.5 ETDRS letters. At week 52, mean NEI VFQ-25 total score improved by + 6.11 ± 11.46, near VA subscale score by + 11.37 ± 18.01, and distance VA subscale score by + 7.33 ± 17.32. At week 52, the mean gain was + 10 ± 8 ETDRS letters [13]. Our results are consistent with those of the AQUA study. Similarly, studies such as RESTORE, RISE, and RIDE highlighted higher score gains in the near vision subscale compared to other subscales [14–16]. The NEI VFQ-25 questionnaire was also utilized in the VIDID and VISTA studies, further supporting our findings of improved near and distance scores with aflibercept treatment [1, 2]. Our study confirmed this pattern, with significant gains in the near vision subscale (+ 20 ± 14%) and distance vision subscale (+ 15 ± 13%).

Previous studies have demonstrated correlations between improvement in BCVA and increased questionnaire scores. At the same time, even with a decrease in BCVA, there was a decrease in the total questionnaire score [17], as well as a significant correlation between NEI VFQ-25 near vision subscale score and BCVA [18]. Our study contributes by affirming direct correlations between near and distance VA values and NEI VFQ-25 subscale and total scores. In contrast, the questionnaire score did not correlate with changes in OCT during treatment.

Despite these contributions, our study has limitations, including a measured timeline in the number of applications rather than weeks, a relatively small sample size with two different diagnoses and treatment regimens, potential influence of the untreated eye on the questionnaire score, and lack of measurement for pupil width or lux levels during near VA monitoring.

## Conclusions

In summary, our study reveals a notable improvement of 4 lines in logMAR optotype for near vision after 8 applications of 2.0 mg/0.05 ml aflibercept to DME and RVO eyes. The significance of monitoring near vision is underscored by the substantial improvement observed in the near vision subscale of the NEI VFQ-25 questionnaire, which started with the lowest scores at baseline. Additionally, we established statistically significant relationships and correlations between BCVA and near VA in relation to both total and subscale scores of the NEI VFQ-25 questionnaire.

## Data Availability

The datasets analysed during the current study are available from the corresponding author on reasonable request.

## Abbreviations

DME: diabetic macular edema
RVO: retinal vein occlusion
BCVA: best corrected visual acuity
VA: visual acuity
AMD: age-related macular degeneration
antiVEGF: anti vascular endothelial growing factor
NEI VFQ-25: National Eye Institute Visual Function Questionnaire
OCT: optical coherence tomography
ETDRS: Early Treatment of Diabetic Retinopathy Study
CRT: central retinal thickness
